# Unraveling the PFOS-NSCLC axis: integrated network toxicology, machine learning, and causal inference identify EIF4EBP1 as a key molecular hub

**DOI:** 10.3389/fpubh.2026.1751125

**Published:** 2026-02-12

**Authors:** Ting Huang, Huaxin Pang, Jundan Wang, Junhua Guo, Keke Hu, Heran Zhou

**Affiliations:** 1 Department of Oncology, Hangzhou TCM Hospital Affiliated to Zhejiang Chinese Medical University, Zhejiang, Hangzhou, China; 2 Chinese Medicine Data Center, China Academy of Chinese Medical Sciences, Beijing, China

**Keywords:** adverse outcome pathway, EIF4EBP1, Mendelian randomization, network toxicology, non-small cell lung cancer, perfluorooctane sulfonate (PFOS), tumor immune microenvironment

## Abstract

**Background:**

Perfluorooctanesulfonic acid (PFOS) is a persistent environmental pollutant with suspected carcinogenic potential; however, the molecular mechanisms driving PFOS-associated non-small cell lung cancer (NSCLC) remain obscure. In particular, the interplay between chemical exposure, oncogenic signaling nodes, and tumor microenvironment (TME) remodeling is poorly defined. This study integrates systems toxicology with multi-omics to elucidate the role of EIF4EBP1 as a mechanistic bridge connecting PFOS exposure to NSCLC pathogenesis.

**Methods:**

We synthesized chemical–protein interactions from toxicological databases (ChEMBL, STITCH, and SwissTargetPrediction) and disease-associated genes to map the PFOS–NSCLC intersection. Robust feature selection, utilizing LASSO and SVM-RFE algorithms, was applied to transcriptomic data from the GSE33532 discovery cohort to identify core targets. Key findings were substantiated through external validation in The Cancer Genome Atlas (TCGA) dataset, including differential expression and survival analyses. Causal associations were investigated via two-sample Mendelian randomization (MR), and the immune landscape was characterized using the CIBERSORT algorithm. Molecular docking simulations and an adverse outcome pathway (AOP) framework were further employed to assess mechanistic plausibility.

**Results:**

Network analysis identified 41 shared targets significantly enriched in PPAR signaling and xenobiotic metabolism. Machine learning consensus prioritized EIF4EBP1 as a critical hub gene. EIF4EBP1 was significantly upregulated in both the discovery (AUC = 0.936) and TCGA validation cohorts. Clinical analysis revealed subtype-specific prognostic value, where high EIF4EBP1 expression correlated with poor survival in lung adenocarcinoma (LUAD) but favorable outcomes in squamous cell carcinoma (LUSC). Immunologically, EIF4EBP1 expression tracked with an adaptive immune-skewed profile, characterized by increased plasma cell and activated CD4 + memory T cell infiltration. MR analysis indicated a potential causal effect of genetically predicted EIF4EBP1 expression on increased LUAD risk (OR = 4.196, 95% CI: 1.209–14.565), but not squamous cell carcinoma. Structural docking confirmed a stable, non-covalent interaction between PFOS and the EIF4EBP1 binding pocket (−7.2 kcal/mol).

**Conclusion:**

This study identifies EIF4EBP1 as a putative molecular initiating node linking PFOS exposure to LUAD susceptibility and immune modulation. The constructed AOP framework suggests a mechanism wherein PFOS-mediated translational dysregulation contributes to subtype-specific carcinogenesis. These findings provide a data-driven rationale for risk assessment and warrant further experimental verification in toxicological models.

## 1 Introduction

Perfluoroalkyl and polyfluoroalkyl substances (PFAS) represent a pervasive class of synthetic fluorinated compounds, renowned for their thermal stability and surfactant properties. Their extreme environmental persistence and resistance to metabolic clearance have led to ubiquitous global distribution and bioaccumulation in human tissues, raising profound public health concerns regarding long-term toxicity (1). Among these compounds, perfluorooctanesulfonate (PFOS) stands out as one of the most extensively produced and studied “forever chemicals,” with well-documented hepatotoxicity, endocrine-disrupting effects, and immunomodulatory potential (2, 3). Concurrently, lung cancer—specifically non-small cell lung cancer (NSCLC)—remains the leading cause of cancer-related mortality worldwide (4). While significant advances have been made in treatment, the role of modifiable environmental risk factors (5), such as chemical exposures, in NSCLC etiology is increasingly recognized yet often under-investigated. Given that the lung is a highly vascularized organ and a primary interface for environmental interaction, it constitutes a plausible site for PFOS bioaccumulation and subsequent toxicity. Nevertheless, the precise molecular mechanisms through which PFOS might influence pulmonary carcinogenesis and tumor progression remain poorly defined.

Current epidemiological evidence linking PFOS exposure to lung cancer risk is suggestive (6, 7) but heterogeneous, with reported associations varying across cohorts and exposure metrics. Such inconsistency likely reflects challenges in real-world exposure assessment, residual confounding (particularly smoking), and co-exposure mixtures common to PFAS research. As a result, epidemiology alone has not yet delineated which NSCLC subtypes or molecular programs are most vulnerable to PFOS-related perturbations, underscoring the need for mechanistic triangulation to strengthen causal interpretation. At a mechanistic level, experimental studies indicate that PFAS can perturb critical cellular processes, including gene expression programs, epigenetic regulation, and xenobiotic transport (8). However, critical knowledge gaps persist. Historically, toxicological research has disproportionately prioritized hepatic and metabolic endpoints, leaving the PFOS-associated molecular landscape in human lung tissue largely uncharted. In particular, it remains unclear whether PFOS preferentially targets genes that are both central to NSCLC biology and linked to metabolic-immune interfaces within the tumor microenvironment (TME).

Mechanistically, PFOS acts as a structural analogue to fatty acids and is a well-established disruptor of lipid metabolism and peroxisome proliferator-activated receptor (PPAR) signaling (9). Emerging evidence suggests that such environmental metabolic perturbations frequently crosstalk with the PI3K-Akt–mTOR axis—a master regulator of cell growth and translational control often hyperactivated in lung adenocarcinomas. Consequently, downstream effectors of this metabolic-translational interface, particularly those governing cap-dependent translation, may represent critical but unverified convergence nodes where PFOS-driven environmental insults synergize with intrinsic oncogenic signaling (10). Yet, whether PFOS exposure specifically intersects with such translational control hubs to drive NSCLC progression and immune remodeling has not been systematically explored.

To address these gaps, we developed an integrated framework combining network toxicology, multi-omics, and genetic causal inference. We hypothesized that PFOS perturbs LUAD-relevant metabolic–translation–immune axes and converges on a limited set of hub genes. Accordingly, we triangulated chemical–protein interaction resources, NSCLC transcriptomics, and large-scale genetic consortia to prioritize core targets, delineate TME infiltration by the CIBERSORT algorithm, and test putative causal directionality via two-sample MR (11). Molecular docking and a putative adverse outcome pathway (AOP) were then used to assess structural and mechanistic plausibility (12). Together, this strategy links PFOS exposure to LUAD susceptibility and immune remodeling, providing a computationally inferred, biologically plausible, and testable hypothesis for future experimental validation and risk-assessment studies.

## 2 Materials and methods

### 2.1 Acquisition of PFOS-associated molecular targets

The canonical SMILES representation of PFOS was obtained from the PubChem database. To systematically identify its potential protein targets, we interrogated three independent toxicological databases: ChEMBL, STITCH, and SwissTargetPrediction, with the organism filter set to *Homo sapiens*. To ensure the reliability of the identified chemical–protein interactions, specific confidence thresholds were applied: for STITCH, only targets with a “combined score” ≥ 0.4 (medium confidence) were retained; for SwissTargetPrediction, targets with a non-zero probability score were included. This multi-database consensus strategy served as an initial reliability filter, prioritizing targets substantiated across different predictive algorithms. The resulting candidate target lists from these resources were consolidated, and duplicate entries were removed to generate a non-redundant set of PFOS-associated molecular targets.

### 2.2 Compilation of NSCLC-associated genes

To ensure a comprehensive compilation of NSCLC-associated genes, we retrieved candidate genes from two complementary databases: the Online Mendelian Inheritance in Man (OMIM), an authoritative resource for curated gene-disease relationships, and GeneCards, an integrative database that aggregates information from multiple sources. This dual-database approach was designed to maximize the coverage of potentially relevant genes. The gene sets from both sources were merged and subjected to identifier standardization. Following the removal of duplicates, a unique, consolidated list of NSCLC-associated genes was established for subsequent intersection analysis with the PFOS targets.

### 2.3 Functional enrichment analysis

To delineate the biological roles and pathways associated with the shared PFOS-NSCLC targets, we performed Gene Ontology (GO) and Kyoto Encyclopedia of Genes and Genomes (KEGG) pathway enrichment analyses using the DAVID bioinformatics platform (13). The analysis comprehensively covered biological processes (BP), cellular components (CC), and molecular functions (MF). Terms with a false discovery rate (FDR)-adjusted *p*-value <0.05 were deemed statistically significant.

### 2.4 Machine learning-based feature selection

To identify robust diagnostic biomarkers from the identified targets, we employed two complementary machine learning algorithms targeting the 41 overlapping PFOS-NSCLC genes within the GSE33532 transcriptomic dataset. The machine learning workflow was designed and reported in alignment with the DOME (Data, Optimization, Model, and Evaluation) recommendations to ensure transparency and reproducibility in supervised ML for the life sciences. The least absolute shrinkage and selection operator (LASSO) regression was implemented using the “glmnet” R package. Genes retaining non-zero coefficients in the majority of iterations were identified as LASSO-selected candidates. In parallel, support vector machine-recursive feature elimination (SVM-RFE) was conducted using the “caret” and “mlbench” packages after feature standardization. An SVM model with a radial basis function (RBF) kernel was trained, utilizing 10-fold cross-validation to rank features based on their predictive contribution. Genes consistently selected by both LASSO and SVM-RFE were defined as the final core targets for downstream validation (14), a strategy designed to minimize overfitting and enhance reproducibility.

### 2.5 Evaluation of differential expression and diagnostic power

Gene expression data from the GSE33532 cohort, comprising paired NSCLC and adjacent normal tissues, were obtained from the Gene Expression Omnibus (GEO) (15) database. Differential expression analysis of the core genes was performed using the Wilcoxon matched-pairs signed-rank test, with the Benjamini–Hochberg procedure applied to control the false discovery rate (FDR) for multiple comparisons. The diagnostic performance of each candidate gene was evaluated by constructing receiver operating characteristic (ROC) curves and calculating the area under the curve (AUC).

#### 2.5.1 Rationale for discovery cohort selection and external validation framework

We prioritized the GSE33532 dataset as our primary discovery cohort based on two rigorous criteria: (i) it was generated using a high-resolution, widely standardized microarray platform (Affymetrix HG-U133 Plus 2.0; GPL570), which minimizes cross-platform technical heterogeneity during feature extraction; and (ii) it features a unique multi-region sampling design (four distinct tumor regions per patient) with matched normal lung tissues. This intra-individual paired design is highly effective at mitigating confounding by inter-individual baseline expression noise, thereby enhancing the reliability of the features prioritized by machine learning. Furthermore, we explicitly utilized this cohort for feature prioritization rather than developing a final diagnostic classifier, a strategy specifically designed to minimize the risk of overfitting and information leakage.

To substantiate the generalizability of the prioritized targets across different sequencing platforms and larger populations, we incorporated an independent external validation framework using The Cancer Genome Atlas (TCGA) database. RNA-sequencing data (log_2_[TPM + 1]) for lung adenocarcinoma (LUAD, *n* = 483), lung squamous cell carcinoma (LUSC, *n* = 486), and their respective normal controls were retrieved via the UCSC Xena platform. In addition to differential expression analysis, we evaluated the clinical significance of the core genes by performing Kaplan–Meier survival analysis on the TCGA cohort. Patients were stratified into high- and low-expression groups based on median levels, and differences in overall survival (OS) were quantified using log-rank tests and hazard ratios (HR) with 95% CIs.

### 2.6 Profiling of tumor immune microenvironment

The composition of immune cell infiltrates in NSCLC and normal tissues was characterized using the CIBERSORT algorithm. This deconvolution method estimates the relative proportions of 22 distinct immune cell types based on the LM22 signature matrix, a validated leukocyte gene signature file. The analysis was performed with 1,000 permutations to assess the statistical significance of the deconvolution results. Only samples with a CIBERSORT *p*-value <0.05 were considered to have accurate immune composition estimates and were retained for downstream analyses. Differences in the abundance of immune cells between groups were assessed using the Wilcoxon rank-sum test. To account for multiple testing across 22 cell types, *p*-values were adjusted using the Benjamini–Hochberg (FDR) method, with an FDR < 0.05 considered statistically significant. Furthermore, Spearman’s rank correlation analysis was employed to investigate the associations between the expression levels of the core genes and the inferred immune cell fractions (16). Correlation analyses across gene–cell pairs were also subjected to FDR correction, highlighting only robust associations (FDR < 0.05).

### 2.7 Two-sample Mendelian randomization analysis

A two-sample MR framework was adopted to infer a potential causal relationship between genetically predicted core gene expression and NSCLC risk (17). Genetic instruments for EIF4EBP1 expression were derived from cis-expression quantitative trait loci (cis-eQTLs) identified by the eQTLGen Consortium (sample size, *n* = 31,684). Summary-level association statistics for the outcomes—lung adenocarcinoma (LUAD) and lung squamous cell carcinoma (LUSC)—were sourced from the FinnGen consortium R12 release.

Genetic variants located within a ±100 kb cis-window of the EIF4EBP1 gene locus and significantly associated with its expression (*p* < 5 × 10^−8^) were considered as candidate instruments, with a minor allele frequency threshold of >1%. Linkage disequilibrium (LD) clumping was performed using a European reference panel from the 1,000 Genomes Project (clumping window = 10 Mb, *r*^2^ threshold <0.01) to ensure instrument independence and reduce residual correlation among instruments. Palindromic SNPs and those unavailable in the outcome datasets were excluded. The strength of each instrumental variable was quantified using the F-statistic, with values >10 indicating a low risk of weak instrument bias. Steiger filtering was applied to verify that the instruments explained more variance in the exposure (EIF4EBP1 expression) than in the outcome (NSCLC).

The primary causal estimate for the analysis involving a single genetic instrument was calculated using the Wald ratio method. For scenarios with multiple valid instruments, the inverse-variance weighted (IVW) method would have been employed as the primary analysis, supplemented by sensitivity analyses including MR-Egger, weighted median, and MR-PRESSO to assess pleiotropy and robustness.

### 2.8 Molecular docking simulations

The high-resolution three-dimensional crystal structures of the core target proteins were acquired from the RCSB Protein Data Bank (PDB). The three-dimensional structure of PFOS was downloaded from PubChem in SDF format and converted to Mol2 format using Open Babel software. Protein structures were prepared for docking by removing all crystallographic water molecules and adding polar hydrogen atoms to optimize protonation states and hydrogen bonding networks. Molecular docking simulations were performed using AutoDock Vina. The grid box parameters for the binding site were defined, and all necessary input files were prepared using AutoDockTools (v1.5.7). The resulting protein–ligand complexes were visualized and rendered using PyMOL (v3.2), and the conformation with the most favorable (lowest) binding energy was selected for detailed interaction analysis (18).

### 2.9 Construction of the adverse outcome pathway

An AOP was systematically developed to delineate the mechanistic sequence linking PFOS exposure to NSCLC pathogenesis. Potential key events (KEs) were identified by analyzing target–phenotype and gene–phenotype networks. Genes substantiated as shared targets, differentially expressed, and directly interacting with PFOS in docking simulations were designated as candidate molecular initiating events (MIEs). The final AOP was constructed by integrating our computational findings with established literature, mapping a plausible causal trajectory from PFOS exposure (MIE) through intermediate KEs (EIF4EBP1-centered translational reprogramming and immune microenvironment remodeling) to the adverse outcome of NSCLC.

## 3 Results

### 3.1 Identification of shared molecular targets in PFOS-NSCLC

**Figure 1:**
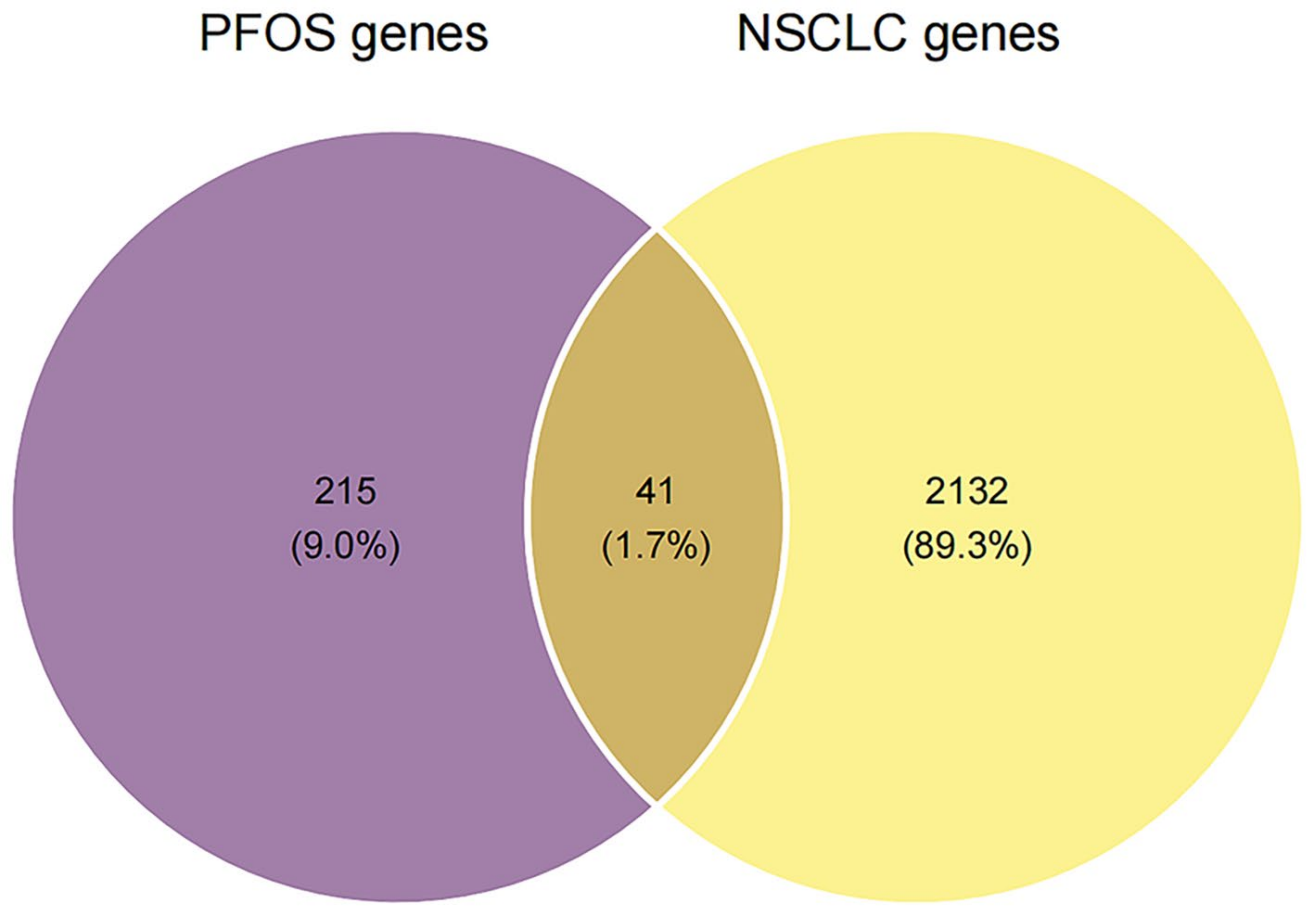
Identification of shared molecular targets between PFOS exposure and NSCLC. Venn diagram illustrating the intersection of putative PFOS-associated targets (blue circle) and known NSCLC-related disease targets (red circle). A total of 41 overlapping genes were identified as potential molecular drivers linking PFOS toxicity to lung carcinogenesis. PFOS targets were predicted using ChEMBL, STITCH, and SwissTargetPrediction; NSCLC targets were compiled from GeneCards and OMIM.

To delineate the molecular landscape linking PFOS exposure to NSCLC pathogenesis, we employed an integrated network toxicology approach. Screening of the ChEMBL, SwissTargetPrediction, and STITCH databases identified 256 putative PFOS targets in *Homo sapiens* (Supplementary Table S1). Concurrently, to establish a robust set of disease-related genes, we compiled NSCLC-associated genes from both the OMIM and GeneCards databases. The merged and deduplicated list contained 2,173 non-redundant genes (Supplementary Table S2). Cross-referencing these datasets yielded 41 overlapping targets, representing high-confidence candidate genes potentially involved in PFOS–NSCLC intersections (Figure 1). To ensure that these overlaps were not merely artifacts of broad NSCLC oncogenic signaling, we verified that each of the 41 genes exhibited documented toxicological associations with PFOS within the chemical–protein interaction databases. Unlike generic NSCLC-associated gene sets, this specific cohort was significantly enriched in PPAR signaling and lipid-sensing modules (Section 3.2), which are canonical hallmarks of PFAS-mediated metabolic disruption. This dual-criteria approach—requiring both evidence of chemical interaction and disease relevance—was implemented to differentiate PFOS-specific mediators from general cancer background pathways and to mitigate the risk of overinterpretation.

### 3.2 Functional enrichment of PFOS-NSCLC overlapping targets

**Figure 2:**
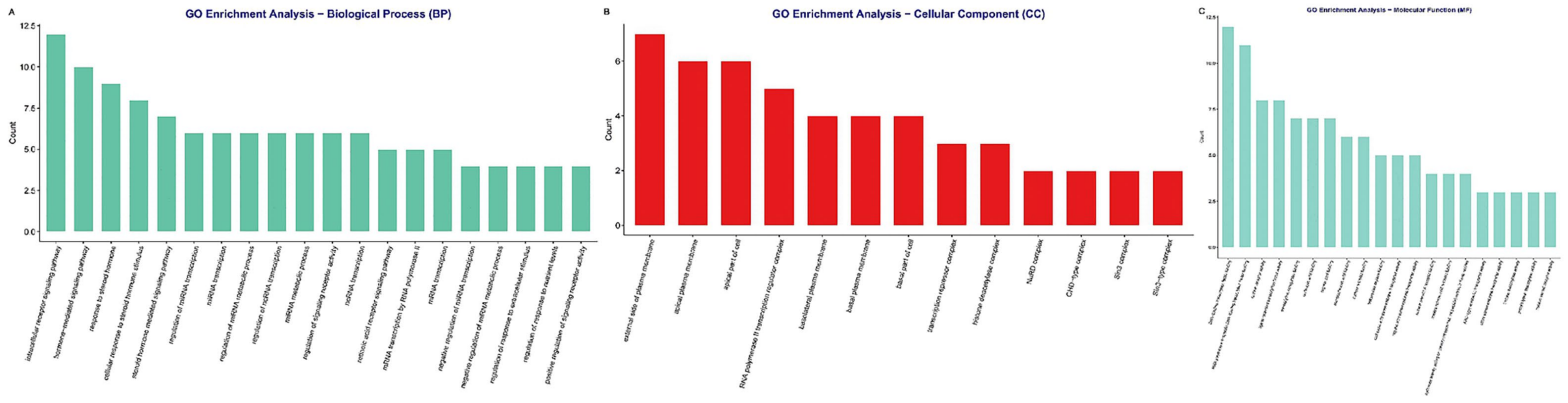
Gene Ontology (GO) functional annotation of the 41 shared PFOS-NSCLC targets. Gene Ontology (GO) enrichment analysis of the 41 shared PFOS-NSCLC targets. The bar charts display the top significantly enriched terms in **(A)** biological processes, **(B)** cellular components, and **(C)** molecular functions. The analysis reveals predominant involvement in intracellular receptor signaling pathways, plasma membrane localization, and nuclear receptor activity.

The GO and KEGG pathway analyses were performed on the 41 shared targets to elucidate their biological significance (Supplementary Tables S3, S4). The GO annotation revealed a predominant enrichment in processes related to intracellular receptor signaling, hormone-mediated responses, and transcriptional regulation (Figure 2A). CC analysis localized these targets primarily to the plasma membrane (particularly the external and apical sides) and within nuclear transcriptional complexes, including RNA polymerase II and histone deacetylase complexes (Figure 2B). MF analysis confirmed their core roles in nuclear receptor activity, ligand-activated transcription factor binding, and histone deacetylase activity (Figure 2C).

**Figure 3:**
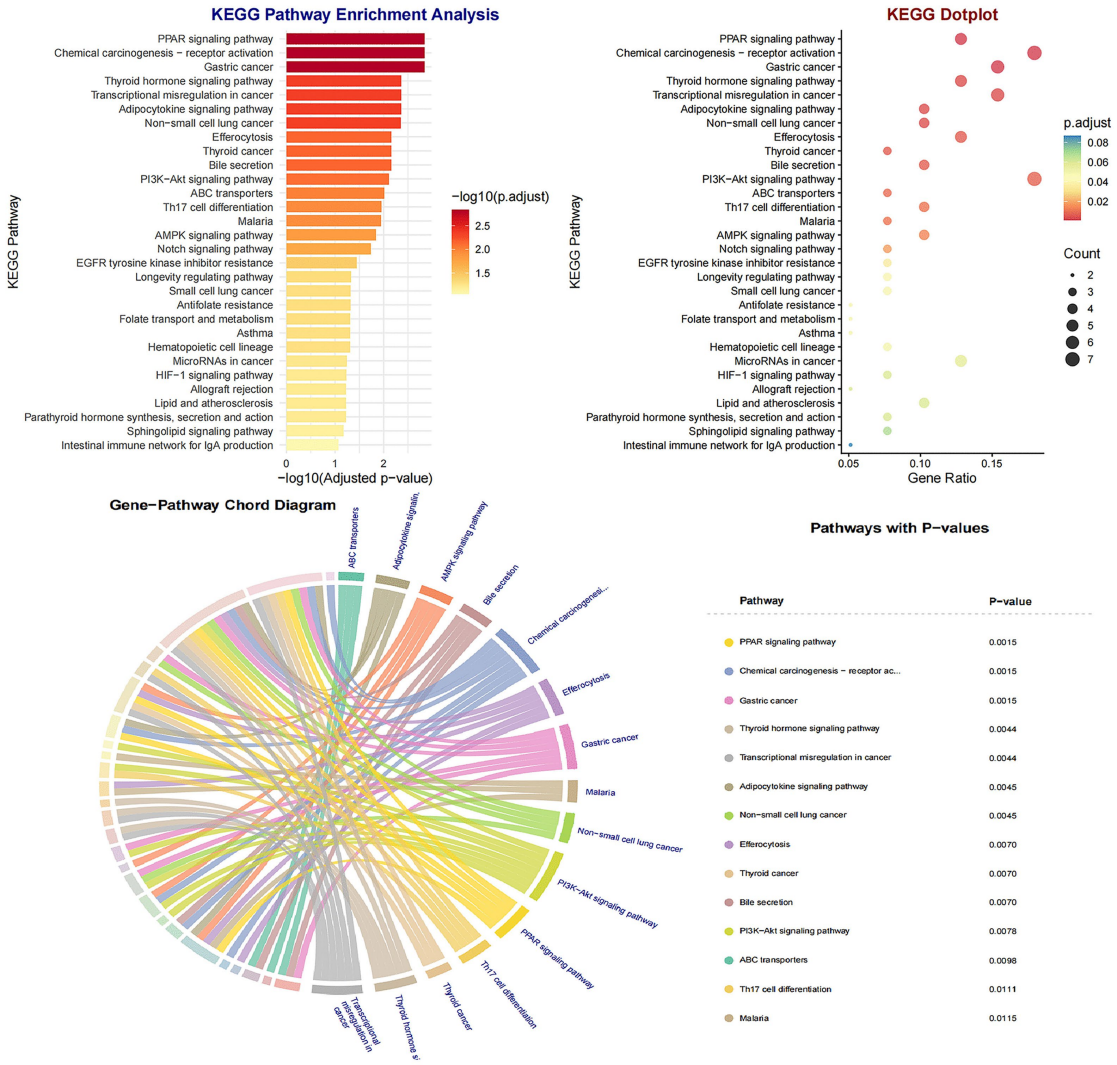
Kyoto encyclopedia of genes and genomes (KEGG) pathway enrichment analysis. Bubble chart showing the top enriched signaling pathways for the shared targets. The size of each bubble represents the number of genes mapped to the pathway, while the color gradient indicates the significance level (adjusted *p*-value). Key pathways include PPAR signaling, chemical carcinogenesis–receptor activation, and transcriptional misregulation in cancer.

The KEGG pathway analysis further underscored significant enrichment in the PPAR signaling pathway, chemical carcinogenesis via receptor activation, and thyroid hormone signaling (Figure 3). Additionally, pathways such as transcriptional misregulation in cancer were also prominently featured. Notably, the mapped genes included key regulators such as CD36, PPARA/G, RXRA/B, VDR, ABCB1, HDAC1-3, and EIF4EBP1. These results collectively suggest that the convergence of PFOS toxicity and NSCLC etiology involves systemic perturbations in lipid-sensing nuclear receptors, epigenetic modulation, and xenobiotic transport mechanisms.

### 3.3 Identification of key diagnostic markers via machine learning

**Figure 4:**
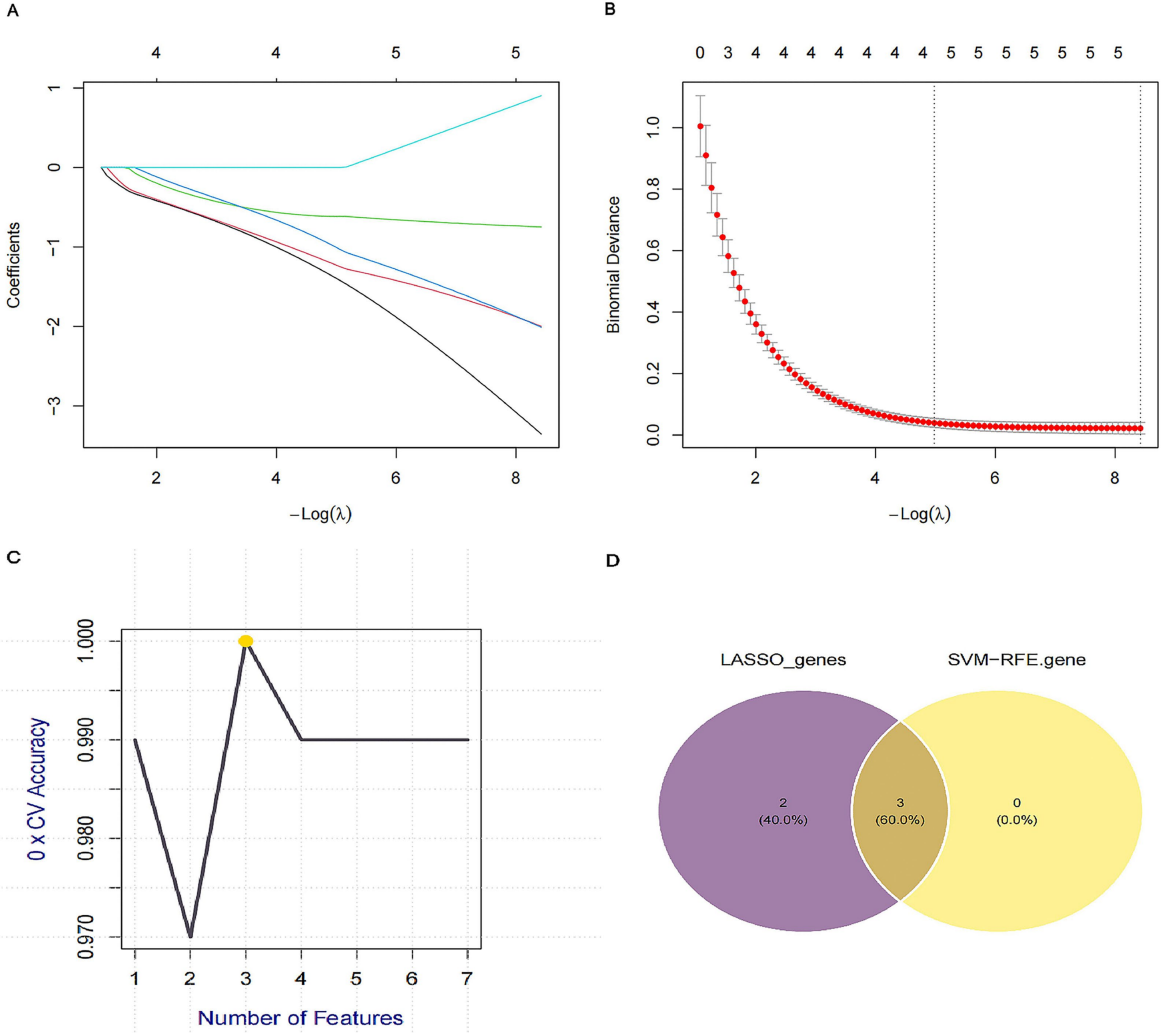
Screening of key diagnostic markers using machine learning algorithms. **(A)** LASSO regression coefficient profiles of the candidate genes. Each curve corresponds to a gene. **(B)** Selection of the optimal penalization parameter (lambda) in the LASSO model using 10-fold cross-validation. The dotted vertical lines indicate the minimum lambda and the lambda within one standard error. **(C)** Feature selection using the support vector machine-recursive feature elimination (SVM-RFE) algorithm to identify the subset of genes with the highest accuracy. **(D)** Venn diagram showing the intersection of candidate genes identified using LASSO (*n* = 5) and SVM-RFE (*n* = 3), yielding three core targets: CD36, ABCB1, and EIF4EBP1.

To pinpoint the most critical diagnostic mediators among the overlapping targets, we applied two machine learning algorithms to NSCLC transcriptomic data. LASSO regression, utilizing 10-fold cross-validation to mitigate overfitting, identified five candidate genes (Figures 4A,B). Simultaneously, SVM-RFE selection isolated three key features (Figure 4C). The intersection of these independent selection methods robustly highlighted three core targets: CD36, ABCB1, and EIF4EBP1 (Figure 4D), which were prioritized for subsequent validation.

### 3.4 Multi-cohort validation of core target expression and clinical significance

**Figure 5:**
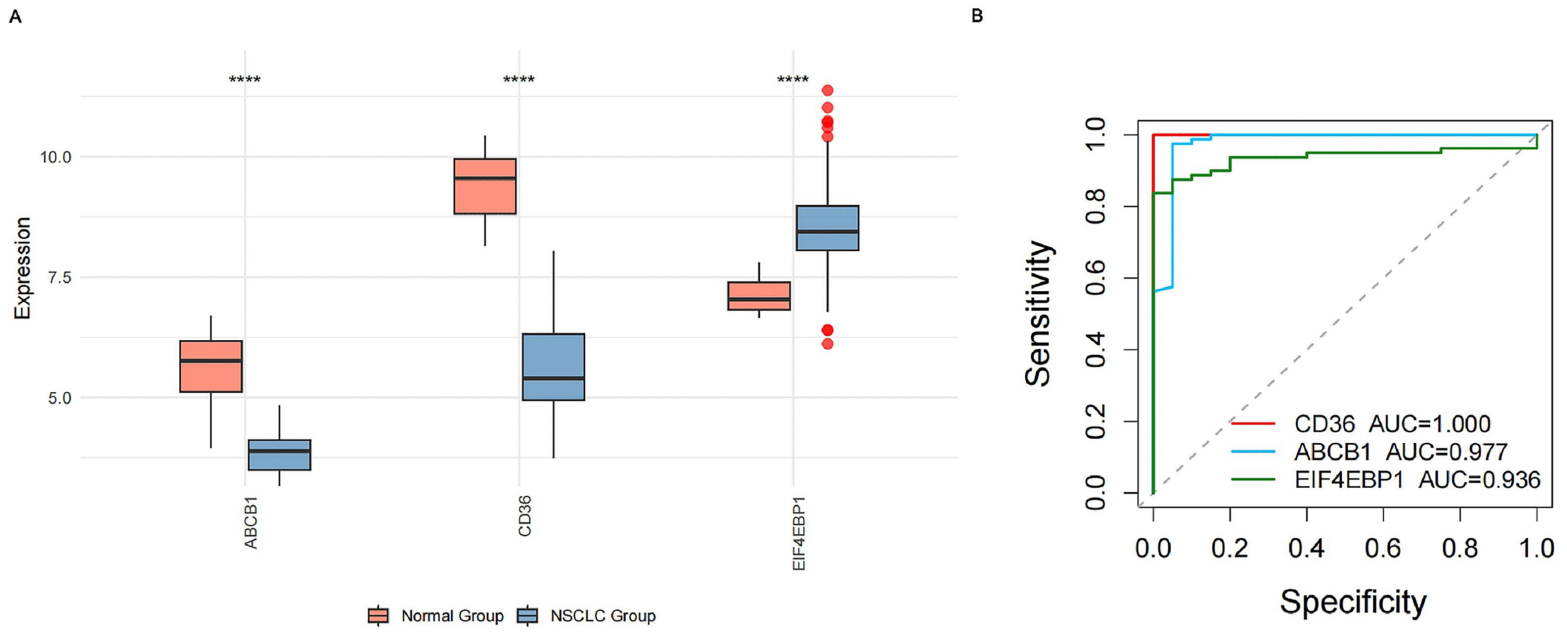
Expression validation and diagnostic performance of core targets in the GSE33532 cohort. **(A)** Boxplots comparing the mRNA expression levels of CD36, ABCB1, and EIF4EBP1 between normal lung tissues (blue) and NSCLC tissues (red). Differences were assessed using the Wilcoxon rank-sum test (*p* < 0.05 was considered significant). **(B)** Receiver operating characteristic (ROC) curves for the three core genes. The area under the curve (AUC) values indicate high diagnostic accuracy for distinguishing tumor from normal tissue.

We first validated the expression profiles of the identified core genes in the GSE33532 discovery cohort. Consistent with the network toxicology screening, EIF4EBP1 was significantly upregulated in NSCLC tissues, whereas CD36 and ABCB1 exhibited marked downregulation (Figure 5A). ROC curve analysis confirmed the robust internal discriminatory performance of the core gene signature, with all markers achieving AUC values >0.90 (Figure 5B). Specifically, EIF4EBP1, a central hub in our integrated analysis, yielded an AUC of 0.936. While these metrics indicate high discriminatory potential within the discovery dataset, they are interpreted as exploratory estimates of diagnostic utility pending independent external validation.

**Figure 6:**
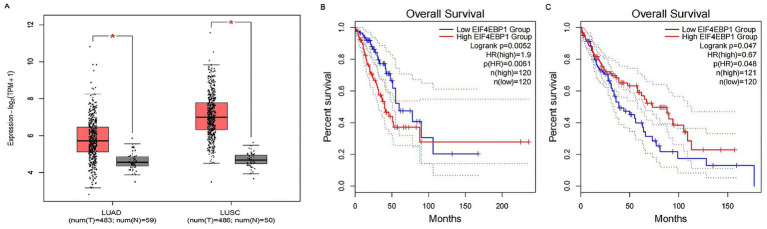
Validation of expression patterns and prognostic significance of the core target EIF4EBP1 in the TCGA cohort. **(A)** Boxplots illustrating the differential mRNA expression of EIF4EBP1 in lung adenocarcinoma (LUAD) and lung squamous cell carcinoma (LUSC) tissues (red) compared to matched normal tissues (blue) from the TCGA database. Statistical significance was determined using the Wilcoxon rank-sum test (*p* < 0.05). **(B,C)** Kaplan–Meier overall survival analysis stratified by EIF4EBP1 expression levels in **(B)** LUAD and **(C)** LUSC patients. Patients were categorized into high (red) and low (blue) expression groups based on the median expression value. Hazard ratios (HRs) and *p*-values (log-rank test) are displayed, revealing that high EIF4EBP1 expression is a risk factor for LUAD (HR = 1.9, *p* = 0.0052) but a protective factor for LUSC (HR = 0.67, *p* = 0.047).

To substantiate the generalizability of these findings, we interrogated the larger, independent TCGA-NSCLC cohorts. Remarkably, the expression patterns observed in the discovery cohort were fully recapitulated in the TCGA datasets: EIF4EBP1 was significantly elevated across both LUAD and LUSC (Figure 6A). Furthermore, survival analysis in the TCGA cohort revealed a striking, subtype-specific prognostic divergence for EIF4EBP1. High expression was significantly associated with diminished overall survival in LUAD (HR = 1.9, 95% CI: 1.2–2.9, *p* = 0.0052; Figure 6B), yet correlated with a paradoxical favorable prognosis in LUSC (HR = 0.67, *p* = 0.047; Figure 6C).

This cross-cohort consistency in expression, coupled with the subtype-specific clinical impact, reinforces the biological significance of our prioritized targets. Among the three core genes, EIF4EBP1 was designated as the primary focus for downstream mechanistic modeling. This prioritization was strategically motivated by its consistent upregulation across platforms (suggesting an oncogenic driver role) and its central position in the mTOR-mediated translational control pathway. Furthermore, its unique structural affinity and subtype-specific causal association identified in Mendelian randomization designated EIF4EBP1 as the most plausible molecular initiating event (MIE) candidate within the PFOS–NSCLC axis.

### 3.5 Correlation between core genes and tumor-infiltrating immune cells

**Figure 7:**
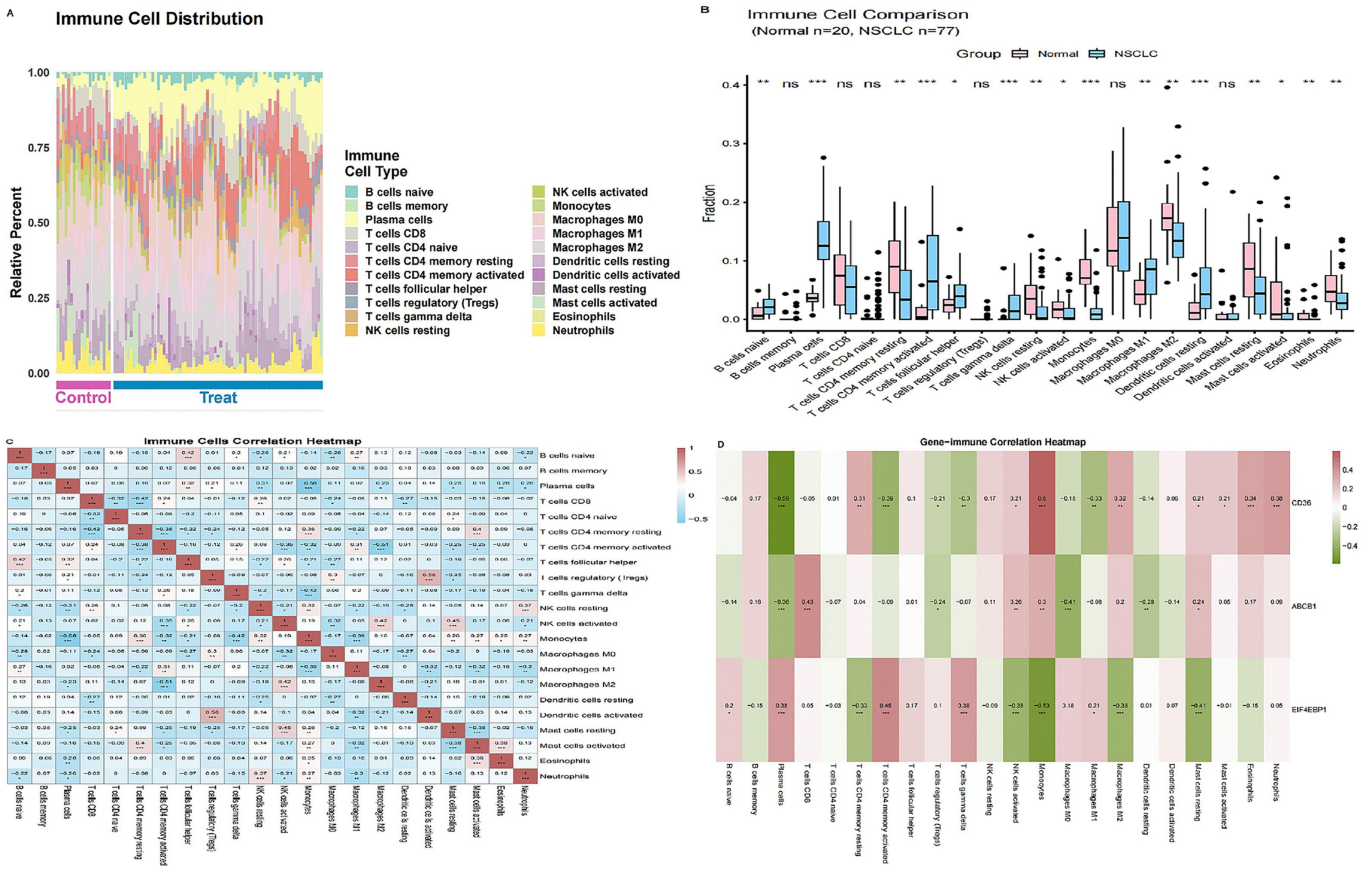
Landscape of tumor-infiltrating immune cells and their correlation with core gene expression. **(A)** Heatmap illustrating the relative abundance of 22 immune cell subsets in NSCLC versus normal lung samples estimated by the CIBERSORT algorithm. **(B)** Violin plots quantifying significant differences in immune cell infiltration levels. **(C)** Correlation matrix of co-infiltrating immune cells, revealing distinct humoral/T-helper and myeloid–granulocytic modules. Blue indicates negative correlation; red indicates positive correlation. **(D)** Heatmap displaying Spearman correlations between the expression of core genes (CD36, ABCB1, and EIF4EBP1) and immune cell infiltration scores. EIF4EBP1 shows a distinct positive correlation with adaptive immune subsets (plasma cells and activated CD4 + T cells). (*p < 0.05, **p < 0.01).

To explore the immune microenvironment, we deconvoluted the abundance of 22 immune cell subsets using the CIBERSORT algorithm. Compared to normal lung tissue, NSCLC samples displayed a marked shift toward an adaptive immune profile, characterized by significantly increased infiltration of plasma cells, activated CD4 + memory T cells, follicular helper T cells, γδ T cells, and M1 macrophages. Conversely, innate effectors such as monocytes, NK cells, neutrophils, and eosinophils were significantly depleted (all *p* < 0.05; Figures 7A,B). Co-infiltration analysis identified two distinct, anticorrelated immune modules: a humoral/T-helper module (plasma cells and activated CD4 + memory T cells) and a myeloid-granulocytic module (monocytes and neutrophils), reflecting divergent immune polarization states within the TME (Figure 7C). These immune shifts primarily reflect tumor-associated remodeling; PFOS relevance is inferred from target overlap and EIF4EBP1-associated patterns rather than direct exposure measurements.

We further examined the specific association between the core genes and these immune subsets (Supplementary Table S5). While CD36 and ABCB1 generally correlated with myeloid and resting lymphocyte subsets, EIF4EBP1 exhibited a strong positive correlation with adaptive immune components—specifically plasma cells, activated CD4 + memory T cells, and M1 macrophages—and a negative correlation with monocytes and M2 macrophages (Figure 7D). This distinct pattern suggests that EIF4EBP1 upregulation in the tumor milieu is closely linked to an adaptive-skewed, yet potentially remodeled and dysregulated, immune microenvironment.

### 3.6 Causal association of EIF4EBP1 with NSCLC risk: a Mendelian randomization analysis

**Figure 8:**
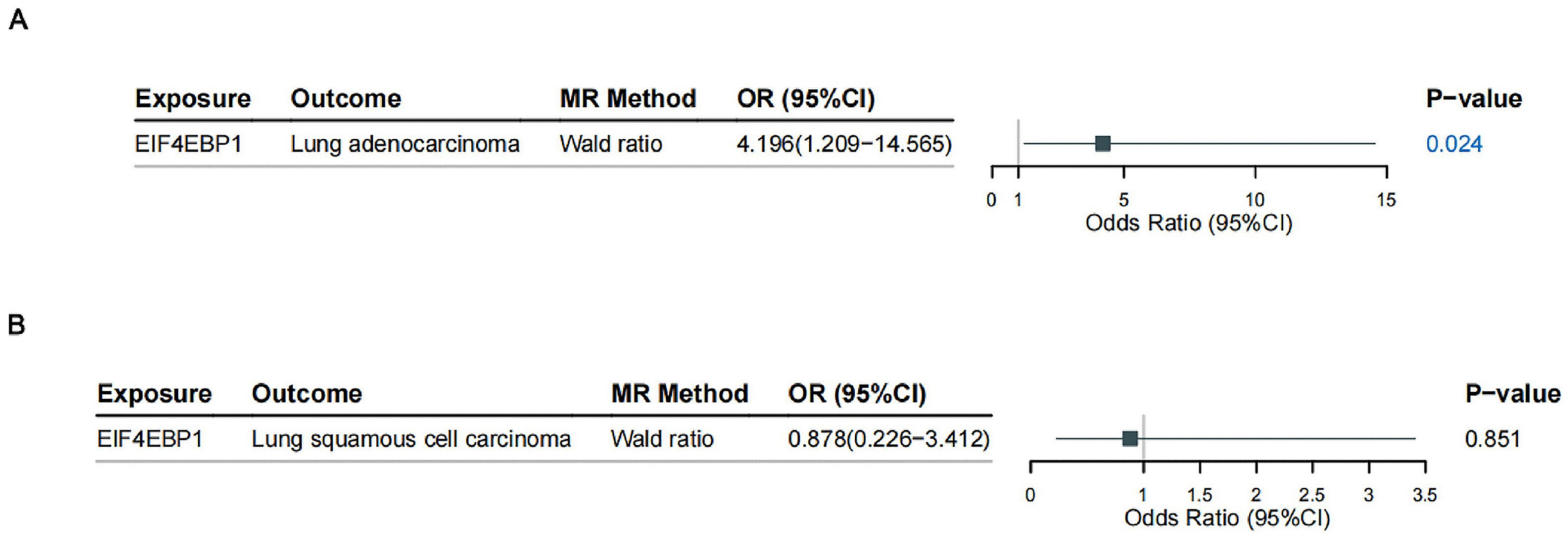
Two-sample Mendelian randomization (MR) analysis of the causal effect of EIF4EBP1 expression on lung cancer subtypes. Forest plots summarizing the causal estimates for the effect of genetically predicted EIF4EBP1 expression on **(A)** lung adenocarcinoma (LUAD) and **(B)** lung squamous cell carcinoma (LUSC). The analysis utilized the *cis*-eQTL SNP rs28565141. The results indicate a significant risk-increasing effect for LUAD (OR = 4.196, *p* = 0.024) but no significant effect for LUSC. OR, odds ratio; CI, confidence interval.

We employed two-sample MR to investigate the causal relationship between genetically predicted EIF4EBP1 expression and NSCLC risk using summary statistics from the FinnGen R12 release (Supplementary Table S6). Using rs28565141 as a valid genetic instrument (F-statistic = 28.622), we found that genetically predicted higher expression of EIF4EBP1 was causally associated with an increased risk of LUAD (β_MR = 1.434, SE = 0.635, *p* = 0.024; OR = 4.196, 95% CI: 1.209–14.565) (Figure 8A). In contrast, no significant causal effect was observed for LUSC (β_MR = −0.130, SE = 0.692, *p* = 0.851; OR = 0.878, 95% CI: 0.226–3.412) (Figure 8B). These findings provide genetic support consistent with a subtype-specific association, implicating EIF4EBP1 as a candidate contributor to LUAD risk.

### 3.7 Molecular docking of PFOS with EIF4EBP1

**Figure 9:**
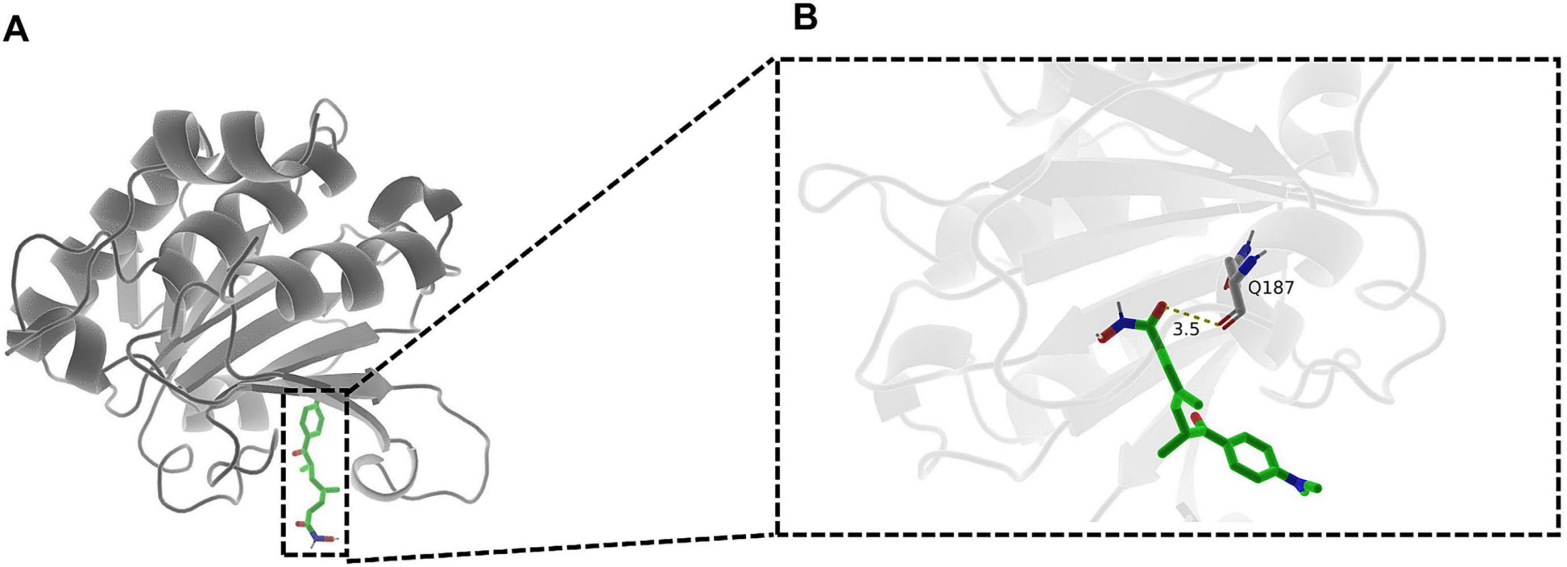
Molecular docking simulation of the PFOS-EIF4EBP1 complex. **(A)** Surface representation of EIF4EBP1 showing PFOS (cyan stick model) binding within a shallow groove defined by α-helices and β-strands. The binding pocket comprises both hydrophobic and polar regions. **(B)** Detailed three-dimensional (3D) view of the binding interface. Key residues interacting with PFOS are labeled (Gln187, Lys88, and Trp89). Dashed lines represent hydrogen bonds or significant polar interactions.

To assess the structural basis of the direct PFOS-EIF4EBP1 interaction, we performed molecular docking simulations. PFOS was predicted to occupy a shallow groove on the EIF4EBP1 surface, adopting an extended conformation stabilized by a mixture of hydrophobic and polar residues (Figure 9A).

**Figure 10:**
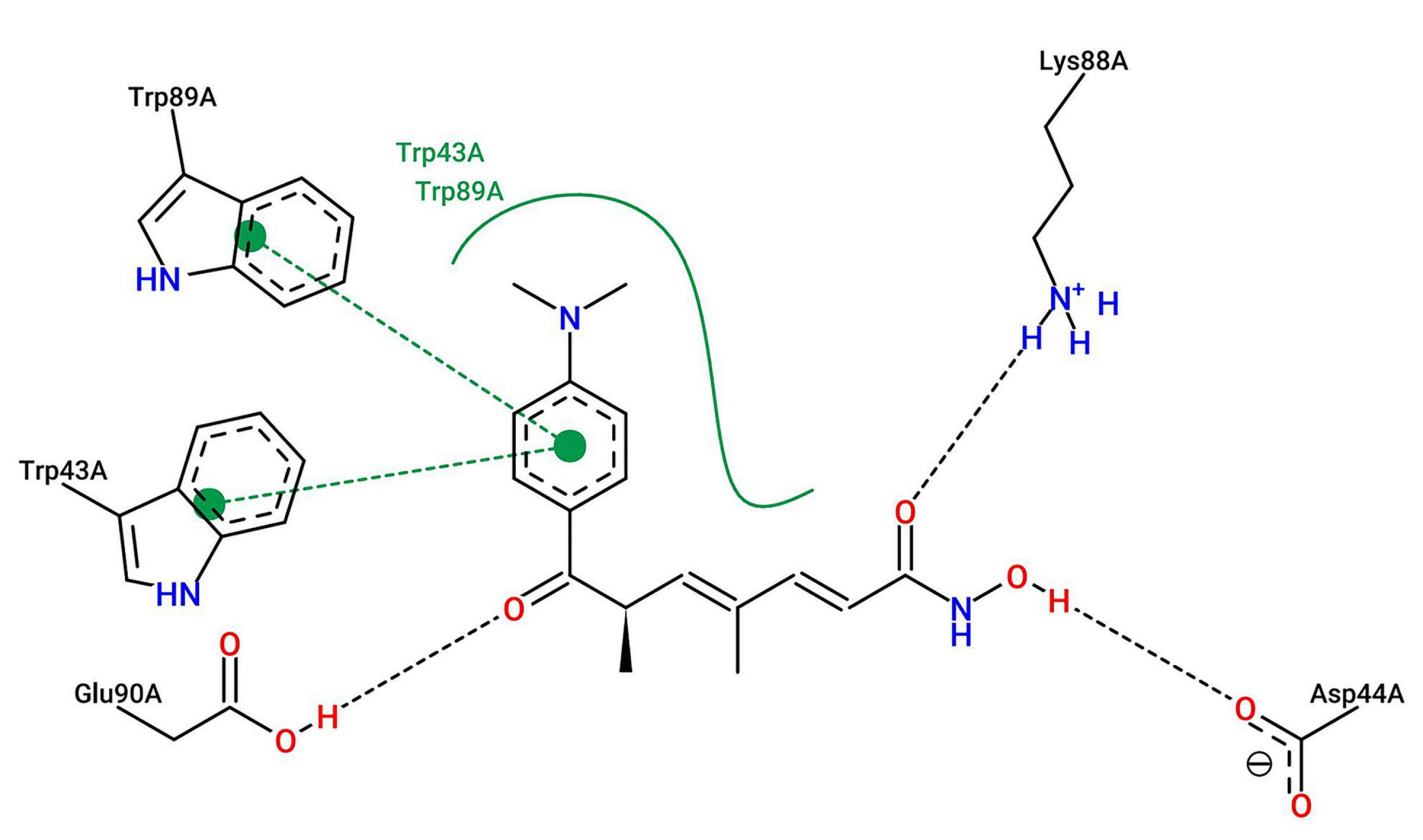
Two-dimensional interaction map of PFOS binding to EIF4EBP1. Diagram illustrating the specific intermolecular forces stabilizing the complex. Green dashed lines indicate hydrogen bonds with distance measurements (Å). Eyelash-like symbols represent hydrophobic contacts. The schematic highlights critical interactions with Asp44A, Lys88A, and Trp89A, confirming a stable non-covalent complex supported by H-bonds and π–π stacking.

Detailed analysis of the binding interface revealed a network of stabilizing interactions (Figures 9B, 10). The terminal polar group of PFOS forms critical hydrogen bonds with the side-chain amide of Gln187, the carboxylate of Asp44A, and the amine of Lys88A (H-bond distances 1.9–3.1 Å). Notably, the interaction with Lys88A exhibits partial salt-bridge character. Beyond polar contacts, the hydrophobic fluoroalkyl tail of PFOS engages in extensive van der Waals interactions and parallel π–π stacking with the indole rings of Trp43A and Trp89A. This cooperative network of hydrogen bonds, hydrophobic contacts, and stacking interactions suggests that PFOS can form a stable, non-covalent complex with EIF4EBP1, potentially modulating its functional state.

### 3.8 Construction of the adverse outcome pathway

**Figure 11:**
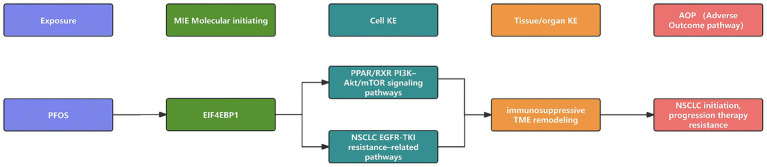
Proposed adverse outcome pathway (AOP) linking PFOS exposure to NSCLC. Schematic representation of the hypothesized mechanistic pathway. The framework progresses from the molecular initiating event (MIE) (PFOS binding to EIF4EBP1 and nuclear receptors) through key events (KEs) (activation of PI3K-Akt/mTOR and PPAR signaling and immune microenvironment remodeling toward an adaptive-skewed but dysregulated state) to the adverse outcome (AO) (NSCLC initiation, progression, and drug resistance). Solid arrows indicate causal relationships supported by this study’s computational findings.

Integrating the findings from multi-omic analyses, we constructed a putative AOP linking chronic PFOS exposure to NSCLC pathogenesis. In this framework, the molecular initiating event (MIE) involves the direct binding of PFOS to core targets such as EIF4EBP1. This interaction is hypothesized to elicit downstream key events (KEs), potentially culminating in NSCLC initiation/progression and therapy resistance. These molecular and pathway-level changes propagate to cellular responses, specifically the remodeling of the tumor immune microenvironment toward a dysregulated adaptive state. Collectively, this cascade of events culminates in the adverse outcome (AO) of NSCLC initiation, progression, and potential therapy resistance (Figure 11).

## 4 Discussion

Widespread exposure to PFOS and its prolonged biological half-life have raised significant concerns regarding its potential carcinogenicity (19). While epidemiological evidence increasingly links PFAS mixtures to various malignancies (20, 21), the association with NSCLC remains under-characterized. By integrating network toxicology, multidimensional transcriptomics, and MR within an AOP framework, this study establishes a “Discovery-Validation” workflow that prioritizes EIF4EBP1 as a putative molecular hub connecting PFOS exposure to NSCLC-related risk signals. Our findings are consistent with the hypothesis that PFOS toxicity extends beyond established hepatic endpoints, potentially converging on lung carcinogenesis through nuclear receptor perturbation, PI3K-Akt/mTOR signaling activation, and immune microenvironment remodeling.

The prioritization of EIF4EBP1 over CD36 and ABCB1 is biologically motivated by its role as a “translational switch.” While CD36 and ABCB1 are primarily involved in xenobiotic transport and lipid uptake, EIF4EBP1 functions as a critical convergence point where oncogenic signaling and environmental stress intersect. This biological centrality makes it an ideal candidate for delineating the mechanistic transition from chemical exposure to malignant transformation. Our network toxicology analysis revealed that shared PFOS–NSCLC targets are significantly enriched in PPAR signaling, lipid metabolism, and ABC transporters. This profile aligns with the known capacity of PFOS to mimic fatty acids, thereby hijacking lipid-sensing nuclear receptors (PPARs) and inducing transcriptional dysregulation (22, 23). Critically, we propose that such metabolic disruption could provide a mechanistic basis for a preferential link between PFOS and LUAD, rather than LUSC (24). This subtype-specific vulnerability is biologically plausible given that LUAD originates from alveolar type II (AT2) cells, which possess a sophisticated metabolic and translational machinery required for surfactant production (25, 26)—a process heavily reliant on lipid homeostasis. EIF4EBP1, as a key regulator of cap-dependent translation, may serve as a critical node where PFOS-induced metabolic stress and intrinsic mTOR signaling hyperactivation converge. In contrast, LUSC is driven by distinct oncogenic trajectories (squamous differentiation) that may be less sensitive to the EIF4EBP1-centered translational axis.

The observed enrichment of PFOS targets in PI3K-Akt/mTOR pathways supports a model wherein PFOS-induced metabolic stress synergizes with mTOR hyperactivation (27). As a downstream effector of mTOR, EIF4EBP1 regulates cap-dependent translation; its phosphorylation promotes the synthesis of oncogenic proteins (28). Consequently, EIF4EBP1 overexpression may represent a critical node where PFOS-driven environmental insults converge with intrinsic oncogenic signaling to fuel adenocarcinoma development.

This mechanistic hypothesis is supported by concordant evidence from genetics, transcriptomics, and structural biology. Two-sample drug-target MR analysis suggested that genetically proxied higher EIF4EBP1 expression is associated with an increased risk of lung adenocarcinoma, but not squamous cell carcinoma. This subtype-specific association is consistent with a potential causal role and aligns with the known predominance of mTOR pathway dysregulation in adenocarcinoma (29, 30). Furthermore, EIF4EBP1 exhibited considerable diagnostic utility (AUC = 0.936) in distinguishing tumor from normal tissue. At the molecular level, docking simulations suggested a favorable binding interaction between PFOS and EIF4EBP1, providing structural plausibility for its designation as a candidate Molecular Initiating Event (MIE) (31, 32).

Beyond tumor cell-intrinsic effects, our analysis highlights a synchronized remodeling of the TME. We observed a shift toward an adaptive immune-skewed profile in NSCLC, characterized by increased infiltration of B cells and M1 macrophages. EIF4EBP1 expression correlated positively with this signature. Although often associated with anti-tumor responses, chronic activation of these pathways in the presence of immunotoxicants such as PFOS can lead to a dysfunctional, pro-tumorigenic inflammatory milieu (33, 34). However, it is important to acknowledge that these immune patterns likely reflect tumor-associated remodeling in general, and any specific contribution of PFOS to these alterations is inferred indirectly.

Our proposed model should be considered alongside alternative interpretations. First, the association between EIF4EBP1 and adaptive immune signatures might be secondary to a more general role for this factor in LUAD aggressiveness or metabolic reprogramming, rather than being a specific consequence of PFOS exposure. Second, the LUAD-specific causal inference from MR analysis could be attributable to the distinct cell-of-origin landscape of this subtype. While epidemiological literature comparing PFOS risks across NSCLC subtypes is currently sparse, our findings align with the emerging consensus that adenocarcinoma is more susceptible to metabolic and endocrine-disrupting toxicants due to its secretory origin and specialized metabolic profile. The strength of this study lies in the multidisciplinary triangulation of evidence across chemical, genomic, and genetic-epidemiological domains. By integrating a rigorous discovery phase in GSE33532 (leveraging its unique multi-region paired design) with an independent validation in the large-scale TCGA cohort, we established a robust chain of evidence connecting environmental exposure to clinical outcomes.

However, several limitations warrant consideration. First, while our machine learning approach adheres to DOME (Data, Optimization, Model, and Evaluation) recommendations to maximize internal and external validity, the feature prioritization is inherently bounded by the initial toxicological screening criteria. Second, although MR provides genetic support for causality, the use of a monogenic instrument for EIF4EBP1 necessitates cautious interpretation regarding potential horizontal pleiotropy. Third, the reliance on bulk transcriptomic data entails inherent limitations regarding tissue heterogeneity and tumor purity. While the divergent correlation patterns observed among our core genes suggest a specific biological linkage, we acknowledge that associations between gene expression and immune abundance in bulk samples can be influenced by the varying proportions of stromal and immune components. The lack of single-cell resolution further precludes the precise localization of EIF4EBP1 expression within specific tumor subclones or distinct immune niches. Finally, the inferred PFOS–EIF4EBP1 interaction is computational; validation in relevant animal models of lung carcinogenesis and large-scale human biomonitoring cohorts is essential. Specifically, biophysical assays such as surface plasmon resonance (SPR) are required to determine the binding affinity between PFOS and the EIF4EBP1 protein. *In vitro* studies in lung adenocarcinoma cell lines should focus on measurable endpoints such as the EIF4EBP1 phosphorylation state and subsequent alterations in cap-dependent protein synthesis. Furthermore, the use of EIF4EBP1-deficient models in lung carcinogenesis studies would be critical to confirm whether the observed adverse outcomes are mechanistically driven by this molecular hub. Therefore, the EIF4EBP1-centered AOP proposed here should be interpreted as a conceptual framework for hypothesis generation rather than a definitive mechanistic map.

## 5 Conclusion

In conclusion, this study constructs a novel, EIF4EBP1-centered mechanistic narrative for PFOS-induced lung toxicity. We propose that PFOS promotes LUAD specifically by exploiting the intersection of lipid metabolic signaling and mTOR-mediated translational control, leading to immune modulation and disease progression. These findings provide a prioritized biological rationale for future experimental toxicology and underscore the need to consider specific molecular subtypes when evaluating the carcinogenic risks of PFAS exposure.

## Data Availability

The original contributions presented in the study are included in the article/Supplementary material, further inquiries can be directed to the corresponding author.
